# Ratiometric pH‐Responsive and Biomimetic Microparticles for Quantitative Monitoring of Phagosomal Acidification

**DOI:** 10.1002/chem.71237

**Published:** 2026-06-08

**Authors:** Sophie Michelis, Héloïse Uhl, Florence Niedergang, Jacques Fattaccioli, Blaise Dumat, Jean‐Maurice Mallet

**Affiliations:** ^1^ Laboratoire CPCV UMR8228, Département de Chimie, École normale supérieure, PSL University, Sorbonne Université CNRS Paris France; ^2^ Institut Pierre‐Gilles de Gennes pour la Microfluidique Paris France; ^3^ Université Paris Cité, Institut Cochin, INSERM CNRS Paris France

**Keywords:** pH sensors, lipid microparticles, hydrosoluble BODIPYs, phagosome maturation, endocytosis

## Abstract

We present a modular biomimetic fluorescent sensing platform designed to precisely interrogate the physicochemical mechanisms governing phagosomal acidification. Phagocytosis is an internalization process for micrometric biological objects and the later stages of phagosomal maturation ‐including acidification‐ play a pivotal role in pathogen destruction and immune signaling. In vitro studies with particulate pathogen models have shown that these degradation pathways depend on the nature of the internalized object as well as on the type of receptors engaged. To alleviate this variability and gain a finer understanding of the phagosome maturation, microparticulate fluorescent pH sensors with chemically controlled composition and cell‐like mechanical properties are thus still lacking. The soft particle‐based biosensors presented herein aim at filling this gap by uniquely integrating (i) cell‐mimicking properties with a fluid and deformable interface, (ii) receptor‐specific uptake and (iii) a ratiometric pH‐responsive fluorescence to quantitatively follow the onset and progression of phagosomal acidification. This versatile platform provides a direct means to couple defined interfacial recognition events to intracellular pH measurements, offering new opportunities to unravel phagosomal physiology.

## Graphical abstract

A series of hydrosoluble BODIPY pH probes allows building biomimetic pH sensors based on micrometric oil‐in‐water droplets. The ratiometric fluorescence output offers quantitative information on the evolution of pH during phagosome maturation.

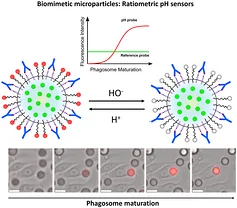

## 1 Introduction

Phagocytosis is a receptor‐mediated internalization and digestion process of objects larger than 0.5 microns leading to the formation of a phagosome. It is accompanied by dynamic variations of the phagosomal pH, which play a central role in regulating degradation, antigen processing, and immune signaling [1, 2]. After internalization, phagosomes undergo progressive acidification driven by the recruitment of vacuolar H^+^‐ATPases (V‐ATPases) and the fusion with endosomal and lysosomal compartments [3]. The resulting decrease in pH activates hydrolytic enzymes and provides an optimal environment for pathogen killing and antigen presentation. Monitoring these changes requires probes that are efficiently internalized by phagocytic cells and that report local acidification with high specificity.

Several fluorescence microscopy–based approaches have been developed to monitor phagosomal maturation and acidification. Freely‐diffusible small molecular probes for pH or enzymatic activity can be used but, in the absence of conjugation to the phagocytosed object, they provide a global view of phagosomal activation dynamics rather than tracking individual internalization events [4, 5, 6]. To achieve more detailed measurements, pH‐sensitive conjugates of bacteria or silica microparticles —typically using commercially‐available probes such as fluorescein isothiocyanate (FITC) or pHrodo red/green— have been employed. These particles are efficiently internalized by macrophages and are compatible with both flow cytometry and fluorescence microscopy analyses [3, 7, 8, 9, 10, 11, 12]. Early work using FITC‐labeled *S. aureus* revealed a rapid loss of fluorescence consistent with phagosomal acidification [3], while studies with FITC‐labeled silica beads reported similar changes occurring with distinct kinetics [8]. In more recent results, IgG‐coated silica beads incorporating pHrodo red provided evidence for a multistage acidification trajectory, involving a lag phase followed by acidification [10]. Using Janus particles, the same research group further demonstrated that the spatial presentation of ligands on the particle surface modulates the kinetics of phagosomal maturation [13].

Two practical issues limit the comparability and resolution of these existing approaches. First, commonly used dyes are not always optimal for multiplexed live phagocytosis imaging and quantitative kinetics: fluorescein/FITC is a turn‐off probe whose fluorescence decreases upon acidification, which hampers efficient detection of phagosome acidification, while pHrodo‐type dyes are turn‐on probes but can retain measurable fluorescence at near‐neutral pH, thus limiting signal to noise ratio. Second, these previous studies highlight that phagosomal maturation is influenced by the physicochemical nature of the target particle, the molecular cues it presents and —as shown previously— by the type of receptor engaged [14, 15]. Bacterial conjugates activate a heterogeneous and poorly‐defined set of membrane receptors and the mechanical properties of solid silica particles strongly differ from that of biological pathogens.

Gaining a precise mechanistic and physiologically‐relevant view of phagosome maturation thus requires micrometric sensors with chemically‐controlled composition and cell‐like mechanical properties to mimic pathogen recognition. To that end, we developed biomimetic targeted lipid microparticles with a ratiometric pH‐dependent fluorescence response to sense phagosomal acidification.

**FIGURE 1:**
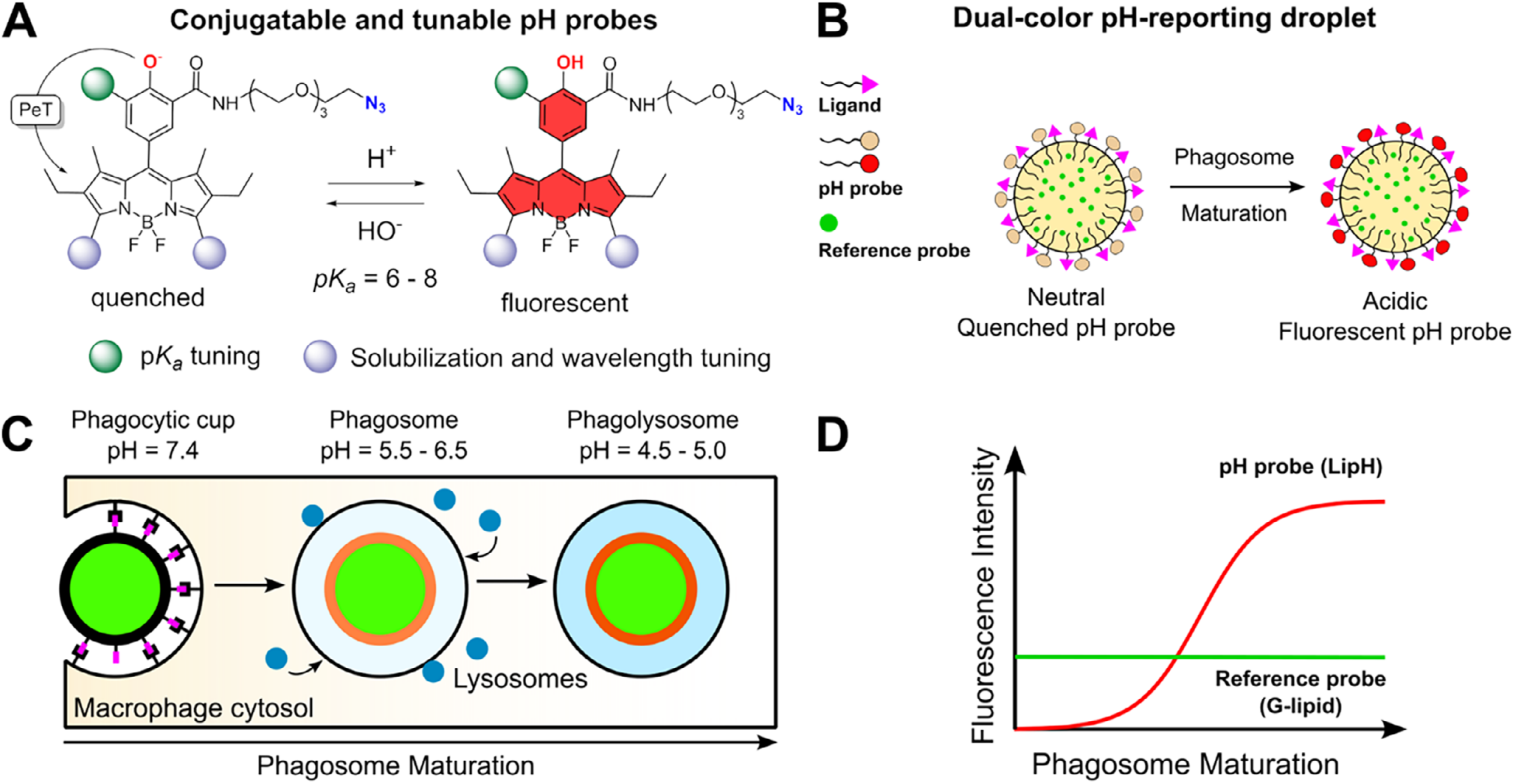
Schematic overview of the design and application of ratiometric pH‐sensitive lipid microparticles for monitoring phagosomal acidification. (A) **Design of hydrosoluble pH probes (BODIpH)**: BODIPY‐derived fluorophores are rendered pH‐sensitive by a phenol group that quenches fluorescence through PeT at neutral pH and restores emission upon protonation in acidic conditions. An azido linker allows bioconjugation and p*K_a_* and emission wavelength can be tuned. (B) **Lipid microparticle formulation**: BODIpH probes are conjugated to phospholipids and incorporated at the droplet surface, together with a pH‐insensitive G‐lipid reference and targeting ligands for receptor‐mediated uptake. (C) **Quantitative pH sensing during phagocytosis**: Following receptor engagement and internalization by macrophages, droplets sequentially encounter phagosomal environments with decreasing pH (7.4 → 5.5–6.5 → 4.5–5). (D) Ratiometric fluorescence readouts enable quantitative measurement of phagosomal acidification dynamics.

We first synthesized a family of water‐soluble, bioconjugatable BODIPY‐derived pH probes (**BODIpH**) with emission wavelengths spanning from yellow to far‐red. These probes display a turn‐on fluorescence from neutral to acidic pH with high dynamic range and p*K_a_* values that can be tuned from 8 to 6, making them well suited for monitoring pH variations during endocytic processes (Figure 1A). Following conjugation to phospholipids, these probes were incorporated onto the surface of micrometer‐sized oil‐in‐water droplets, together with biotinylated ligands allowing opsonization with anti‐biotin immunoglobulin G (IgG) (Figure 1B) [16, 17]. In contrast to solid silica particles, the fluid and deformable interface allows the phagocytic ligands to laterally cluster, better reproducing cell–cell interactions and enabling efficient recognition even by low‐affinity ligands such as monosaccharides that target lectin receptors [17, 18, 19, 20]. A pH‐agnostic lipophilic dye was also encapsulated inside the droplets to provide an internal reference for ratiometric quantification of the phagosomal pH. The design thus combines controlled surface functionalization with targeting ligands to trigger phagocytosis and fluorescent pH probes and the resulting lipid microparticles were phagocytosed by RAW 264.7 macrophages through Fcγ receptors engagement, enabling the measurement of phagosomal acidification over time during the maturation stage (Figure 1C,D).

## 2 Experimental Section

### 2.1 Synthetic Procedures

Detailed synthetic procedures and analysis are available in the supplementary information.

### 2.2 Measurement of pH‐Dependent Fluorescent Properties of BODIpH Probes

Fluorometric pH titrations curves were determined by measuring the fluorescence intensity of the probes at various pH by fluorescence spectroscopy. We used stock solution of the probes at 1 mM in DMSO. Britton‐Robinson buffers of various pH were made by mixing various amount of 40 mM boric acid, 40 mM acetic acid and 40 mM phosphoric acid solutions and adjusting the solutions with hydrochloric acid or sodium hydroxide. For **Yellow BODIpH 1** and **2** the measures were performed in 70% Britton‐Robinson buffer and 30% acetonitrile whereas for **Red** and **fr BODIpH** series the measures were performed in fully aqueous Britton‐Robinson buffers. The fluorescence of each probe was measured three times in each pH buffer. To determine the p*K_a_*, the integrated fluorescence intensity was plotted against pH and fitted using Hill1 equation in Origin 2018 software.

Solubility limits in water were determined by measuring the absorbance of the probes at various concentrations in water. We used stock solution of the probes at 1 mM in DMSO. The absorbance at the maximum absorption wavelength for each solution was plotted against the concentration and fitted to a linear model.

### 2.3 Lipid Droplets Formulation

Droplets are fabricated with a Shirasu Porous Glass (SPG) membrane emulsification device. An hydrophilic‐treated microporous membrane is immersed into the continuous phase of Poloxamer 188 (Sigma‐Aldrich, Ref.: P2443) at 15% w/w to remove the trapped air from the pores. The dispersed phase consisting in Lipiodol (Guerbet, (CAS no. 8002‐46‐8)) is inserted in a tank connected to a pressure valve. A pressure is applied to force the dispersed phase through the micrometer pores. At a critical pressure, droplets form, reach the continuous phase and are stabilized by surfactants. The resulting emulsion is continuously stirred during the process. Droplet's diameters depend on the pore size of the membrane. For the following experiment, a membrane of 2.1 µm was used to obtain droplets with well‐controlled size diameter  of 8 ± 0.8 µm. The emulsion is stocked out of light at a temperature of 12°C in a Peltier‐cooled cabinet.

### 2.4 Droplets Functionalization

The volumes used for the surface functionalization are expressed as volume equivalent. One equivalent (eq.) corresponds to the volume of molecules necessary to cover the total area of a population of droplets with a compact monolayer of molecules [16].

The functionalization of droplets was performed according to the following procedure: 10 µL of 8 µm‐diameter droplets (i.e. 16 millions droplets) in Poloxamer 188 (Pluronic F68, Sigma‐Aldrich), at 15% w/v are placed in a microtube (Axygen, 0.6 mL Microtubes, “Maximum recovery”, Ref.: MCT‐060‐L‐C) and are diluted with phosphate buffer (pH = 7.1, 20 mM) supplemented with Tween20 at the CMC (0.007% w/v) in a way to reach a volume of 200 µL. The diluted emulsion was centrifuged at 2000 rpm for 30 sec. The continuous phase was removed and 195 µL of PB/Tween20 CMC were added again. This washing step was repeated 3 times. After the last rinsing step, only 10 µL of the emulsion volume remain. Then, 20 eq. of DSPE‐PEG(2000)‐Biotin (Avanti Lipids, Ref.: 880129P‐10 mg) (i.e. 2.6 µL for a concentration of 10 mg/mL) and 2.5 µL of G‐Lipid (at 40 µM in DMSO) were added, followed by DMSO to reach a volume of 20 µL of polar solvent. Then the mix was completed with PB/Tween20 CMC to have a total volume of 200 µL. After 30 min rotation in the dark, droplets were washed 3 times with PB/Tween20 CMC. After the last rinsing step, all the supernatant is discarded. Then, 0.5 eq. of IgG anti‐biotin (Jackson Immunoresearch, AMCA‐conjugated IgG Fraction Monoclonal Mouse Anti‐Biotin, Ref.: 200‐152‐211, lot number: 109881) (i.e. 3.9 µL at 0.85 mg/mL) were added to the droplets and the volume is completed with 50 µL PB/Tween20 CMC. The mixture was then placed on a rotary shaker for 30 min in the dark and was washed again 3 times.

Finally, to functionalize the droplets with the pH‐probe, 0.5 eq. of the LipH (i.e. 3 µL for a concentration of 0.4 mg/mL) was added, followed by DMSO to reach a volume of 20 µL of polar solvent, and completed with PB/Tween 20 CMC to have a total volume of 200 µL.

After 30 min rotation in the dark, droplets were washed 3 times with PB/Tween 20 CMC and were ready to be put in contact with macrophages.

### 2.5 Fluorometric pH Titrations Curves for the Functionalized Droplets

Fluorimetric pH titrations curves were determined by measuring the mean fluorescence intensity of the droplets in cell culture medium of various pH by fluorescence microscopy (spinning‐disk inverted microscope Leica DMi8). The pH of the solution was adjusted by addition of NaOH or HCl and controlled with a pH‐meter.

### 2.6 Cellular Fluorescence Imaging

Phagocytic assays were performed in RAW 264.7 macrophage cell line (RRID:CVCL_0493, line established from a tumor induced by Abelson murine leukemia virus, gender: male, strain: BALB/c) transfected with LifeAct mCherry. Cells are grown in 100 mm diameter Petri dishes (Falcon, Ref.: 353003) at 37°C and in a humidified atmosphere containing 5% of CO_2_. Macrophages are plated in complete medium consisting of RPMI Glutamax  (Gibco, Ref.: 61870‐010) supplemented with 10% Fetal Bovine Serum (Gibco, FBS One Shot, Heat Inactivated, Ref.: A3840402), 10 mM HEPES (Gibco, Ref.: 15630‐056), 1 mM sodium pyruvate (Gibco, Ref.: 11360‐070), 50 µM 2‐Mercaptoethanol (Gibco, Ref.: 31350‐010) and 2 mM L‐glutamine (Gibco, Ref.: 25030‐024). The expression of Lifeact_mCherry fluorescent protein was maintained by intermittent addition of 4 µg.mL^−1^ of puromycin (Gibco, Ref,: A11138‐03). Cells used for experiments were between passage 11 and 20.

24 h before experiments, cells were detached with a scraper (Costar, Ref.: 3008) and seeded in new medium on a 24 well glass plates (Cellvis, Ref.: P24‐1.5H‐N) to reach a concentration of about 2,5.10^5^ cells/well. Just before imaging, cell medium was replaced by the same medium free of FBS and red phenol (Gibco, Ref.: 11835030). Imaging experiments were performed with a spinning‐disk inverted microscope (Leica DMi8). To study internalization, about one droplet for two cells was used. After waiting 5 min, the sample was imaged live at 37°C and under controlled‐atmosphere (5% CO_2_). Images were recorded with MetaMorph (Molecular Device). The steam mode with the following parameters were used:

Z‐steps of 3 µm
For the pH‐probe LipH: *λ*_ex_ = 640 Nm/*λ*_em_ = 685±40Nm
For the F‐actin: *λ*_ex_ = 561 Nm/*λ*_em_ = 607±36 Nm
For the G‐Lipid: λ_ex_ = 488 Nm/*λ*_em_ = 525± 30 Nm
For the IgG: *λ*_ex_ = 405 Nm/*λ*_em_ = 525± 30 Nm

For Bafilomycin control experiments, **c**ells were pretreated with Bafilomycin A1 (Sigma‐Aldrich, Ref. 196000–10UG) at 200 nM for four hours. Twenty minutes prior to imaging, the culture medium was replaced with the same medium free of FBS and red phenol, supplemented with 100 nM LysoTracker green and 200 nM Bafilomycin A1. Cells were imaged directly in this treatment medium.

## 3 Results and Discussion

### 3.1 BODIpH Probes: Hydrophilic pH Probes With Tunable Emission Wavelengths

Given the central physiological role of pH, a wide range of fluorescent probes has been developed for its measurement [21]. Most of them are based on rather lipophilic neutral or cationic probes that readily permeate the plasma membrane and enable intracellular pH sensing. Following conjugation to biomolecules or particles such lipophilic probes can lead to aggregation and quenching, which can be harnessed as a useful sensing mechanism [22, 23]. However, in the frame of this project, aggregation on the surface of the particle may lead to quenching or interfere with environmental pH sensing and hydrosoluble probes with limited aggregating ability are thus necessary. Phagocytosis imaging assays frequently require multiplexed fluorescence experiments to visualize additional cellular components —such as actin, receptors, or endosomal markers— alongside exogenous pH sensors. Extending the spectral range of pH‐sensitive probes toward the far‐red region is therefore essential to enable efficient spectral separation and multicolor imaging. Considering these requirements, commonly used probes for phagocytic assays, such as fluorescein (FITC) or pHrodo red and green, remain suboptimal: fluorescein exhibits a turn‐off response and low emission wavelength, while pHrodo dyes display p*K_a_* values around 6.8, resulting in residual fluorescence at neutral pH and a reduced signal‐to‐noise ratio. To address these limitations, we set out to design new hydrosoluble pH probes termed **BODIpH** with tunable optical properties and p*K_a_* values, suitable for particle surface functionalization and multiplexed imaging of phagocytosis (Figure 1A).

**FIGURE 2:**
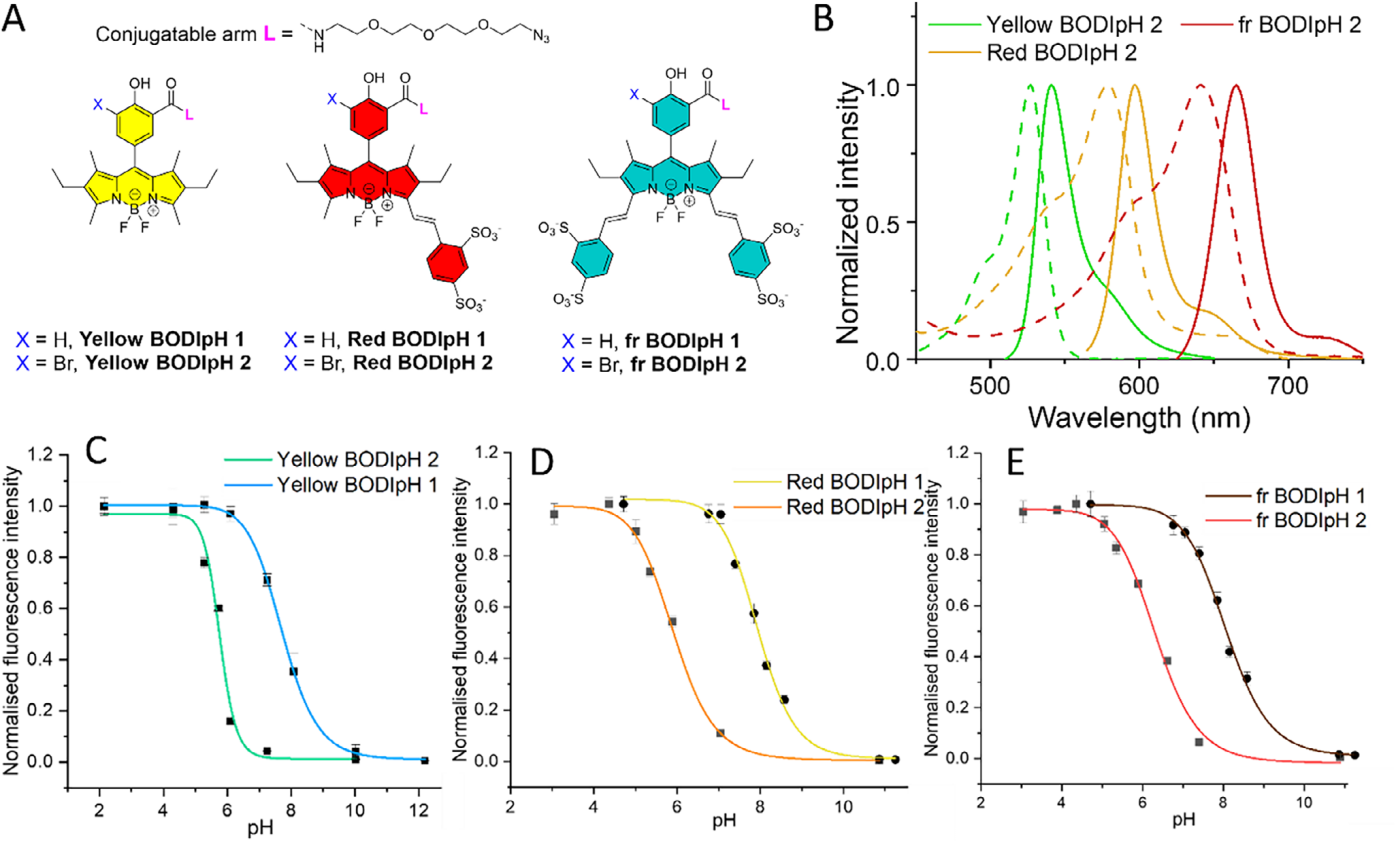
Design and photophysical properties of **BODIpH** probes. (A) Chemical structures of the six **BODIpH** derivatives. (B) Normalized absorption (dashed lines) and emission (solid lines) spectra. Fluorometric pH titration curves for (C) **Yellow BODIpH**, (D) **Red BODIpH**, and (E) **fr BODIpH**. Titrations were performed in aqueous Britton Robinson buffers, except for the Yellow series where 30% acetonitrile was added. Graph display the mean + SD of two independent replicates.

The core scaffold of the **BODIpH** probes is an ethyl‐BODIPY structure (**Yellow BODIpH**) (Figure 2 and Table 1). The pH sensitivity stems from a photoinduced electron transfer (PeT) with a phenol moiety in the meso position of the BODIPY: PeT between the phenolate and the fluorophore quenches the fluorescence that is restored upon protonation creating an on/off emission trigger actuated by protons (Figure 1A) [24, 25, 26]. An amide function in ortho position of the phenol reduces the p*K*_a_ of the phenol by approximately two pH units (pKa ≈ 8, BODIpH series 1) thanks to hydrogen bonding [27]. It was also used to introduce a conjugatable handle containing an azide allowing biorthogonal conjugation to biomolecules or particles. Addition of an electron‐withdrawing bromine atom further decreases the pKa down to around 6 (BODIpH series 2). The synthetic procedures are presented and discussed in the supplementary material (Schemes S1 and S2; Table S1).

**TABLE 1:** Photophysical properties of the BODipH probes.

	pH	*λ*_abs_ (nm)	ε (cm^−1^mol^−1^L)	*λ*_em_ (nm)	Φ_F_ ^a^	p*K*_a_ ^b^	pH sensing range	Full dynamic range	Endocytosis Dynamic range (pH4.5/pH7.5)
Yellow BODIpH 1	6.1	524	74000	538	0.74	7.7 ± 0.01	6–10	170	NA
	10.8	520	76000	538	0.06				
Yellow BODIpH 2	3.8	527	56000	541	0.85	5.8 ± 0.08	5–6.5	90	64
	7.4	522	56000	540	0.05				
Red BODIpH 1	6.1	577	36000	594	0.55	7.9 ± 0.05	6.8–9.6	80	NA
	10.8	570	32000	594	0.04				
Red BODIpH 2	3.8	578	30000	597	0.40	5.9 ± 0.06	4.5–7.4	102	17
	7.4	574	33000	597	0.02				
fr BODIpH 1	6.1	639	78000	662	0.15	8.1 ± 0.05	6.8–9.7	70	NA
	10.8	632	70000	662	0.002				
fr BODIpH 2	3.8	642	70000	665	0.15	6.3 ± 0.08	4.8–7.8	150	10
	7.4	637	75000	665	0.008				

Maximum absorption (*λ*_abs_) and emission (*λ*_em_) wavelength, molar extinction coefficient (ε), fluorescent quantum yield (Φ_F_).

^a^ Fluorescence quantum yields were measured in the most acidic pH indicated in the table for each probe.

^b^ Fitting result and standard deviation of the data presented on Figure 2C‐E.

The BODIPY scaffold was chosen for its good photophysical properties and easily tunable emission wavelength. Red (**Red BODIpH**, *λ*_em_  =  597 nm) and far‐red (**fr BODIpH**, *λ*_em_ = 665 nm) emitting derivatives were obtained by extending the electronic conjugation on position 3 and 5 with sulfonated styryl moieties (Figure 2A and B). BODIPYs display robust fluorescent properties but are intrinsically highly lipophilic and different strategies have been implemented to increase their solubility in aqueous media [28, 29, 30, 31]. Here the introduction of sulfonated moieties not only shifts the emission wavelength but also make them hydrosoluble to enable pH sensing at the interface of the particles with the aqueous environment without interference from aggregation phenomenon [32].

Absorption measurements conducted within the concentration range of 1–20 µM revealed that both the **Yellow BODIpH** and **Red BODIpH** series observe the Lambert–Beer law up to approximately 15 µM, whereas the **fr BODIpH** series exhibit no detectable solubility limit (Figure S1). Despite their apparent solubility, **Yellow BODIpH 1** and **2** display markedly reduced fluorescence in aqueous media compared to their sulfonated red and far‐red analogues, indicative of aggregation‐induced quenching. The use of aqueous buffers supplemented with 30% acetonitrile allowed recovering the fluorescence and performing the photophysical characterization (Figure S2). The moderate solubility observed for the Yellow series, which lacks solubilizing substituents apart from a short PEG chain, aligns with the intrinsic lipophilicity commonly associated with BODIPY derivatives. The red and far red **BODIpH** series could be characterized in fully aqueous media.

The extinction coefficients are high (approx. 60 000 cm^−1^mol^−1^L) and similar for the **Yellow** and **fr BODIpH** series but are decreased for the **Red BODIpH** series (approx. 30 000 cm^−1^mol^−1^L, Table 1). The extension of conjugation leads to a decrease of quantum yield due to the increased flexibility of the styryl branch that favors non radiative decay (φ_F_ around 80% for the yellow series, 50% for the red series, 15% for the far‐red series, Table 1). Overall, the brightnesses remain very satisfactory, especially for fully hydrosoluble BODIPYs with far red emissions [33, 34].

The photophysical and pH‐sensing properties summarized in Figure 2; Table 1; Figure S3 show that all 6 probes display a pH‐sensitive emission with excellent dynamic ranges (70 to 200‐fold fluorescence increase in fluorescence intensity between high and low pH conditions). The p*K_a_* is mostly governed by the structure of the phenol group and regardless of the emission wavelength of the fluorophore part similar values are obtained for **BODIpH** series 1 and 2. A slight p*K_a_* increase of about 0.5 pH unit is nonetheless observed upon increase of the fluorophore conjugation for the two series (Table 1). While these results provide insights into the design of tunable fluorescent pH probes, only the probes from the second series with p*K_a_* values around 6 are useful to sense pH during endocytic processes. In addition to the full dynamic range between the most acidic and basic values, the dynamic range between pH 4.5 and pH 7.5 was also calculated for series 2 as it corresponds to the relevant pH range for endocytosis. **Red** and **fr BODIpH 2** display gradual pH transitions that slightly limit their dynamic range compared to **Yellow BODIpH 2** but leads to a wider pH sensing range between 4.5 and 7.4 that is perfectly suited to monitor pH during endocytic processes such as phagocytosis. Most pH probes dedicated to endocytic processes are limited to narrower sensing ranges of two pH units centered around p*K_a_* [28, 35, 36].

### 3.2 Formulation of Ratiometric pH‐Sensitive and Targeted Fluorescent Microparticles

We previously reported the use of micrometer‐sized oil‐in‐water emulsion droplets as biomimetic particles to study phagocytosis. These droplets can be readily functionalized at their surface with phagocytic ligands such as immunoglobulin G (IgG) or mannose and are efficiently recognized and internalized by macrophages in a receptor‐selective manner [17, 18, 19]. The properties and applications of these droplets in phagocytosis were previously thoroughly characterized and they have excellent biomimetic properties as phagocytic targets compared to solid particles. When the droplet surface is not saturated with ligands such as IgGs, clusters can form at the cell–droplet contact zone which mimics ligand mobility on a cell membrane and has been shown to enhance FcγR signaling [17, 37]. The mechanical properties of the droplets depends on their composition and size. The 8 µm droplets made out of lipiodol used herein are mechanically deformable with an interfacial tension of approximately 1.8 mN/m corresponding to an apparent stiffness of about 4–5 kPa (Figure S5A,B) [20]. These mechanical properties fall within the range reported for soft biological targets and for immune cell mechanosensitivity [38]. Building on these observations and previous results, we adapted this platform to incorporate pH‐sensitive probes for monitoring phagosomal maturation.

**FIGURE 3:**
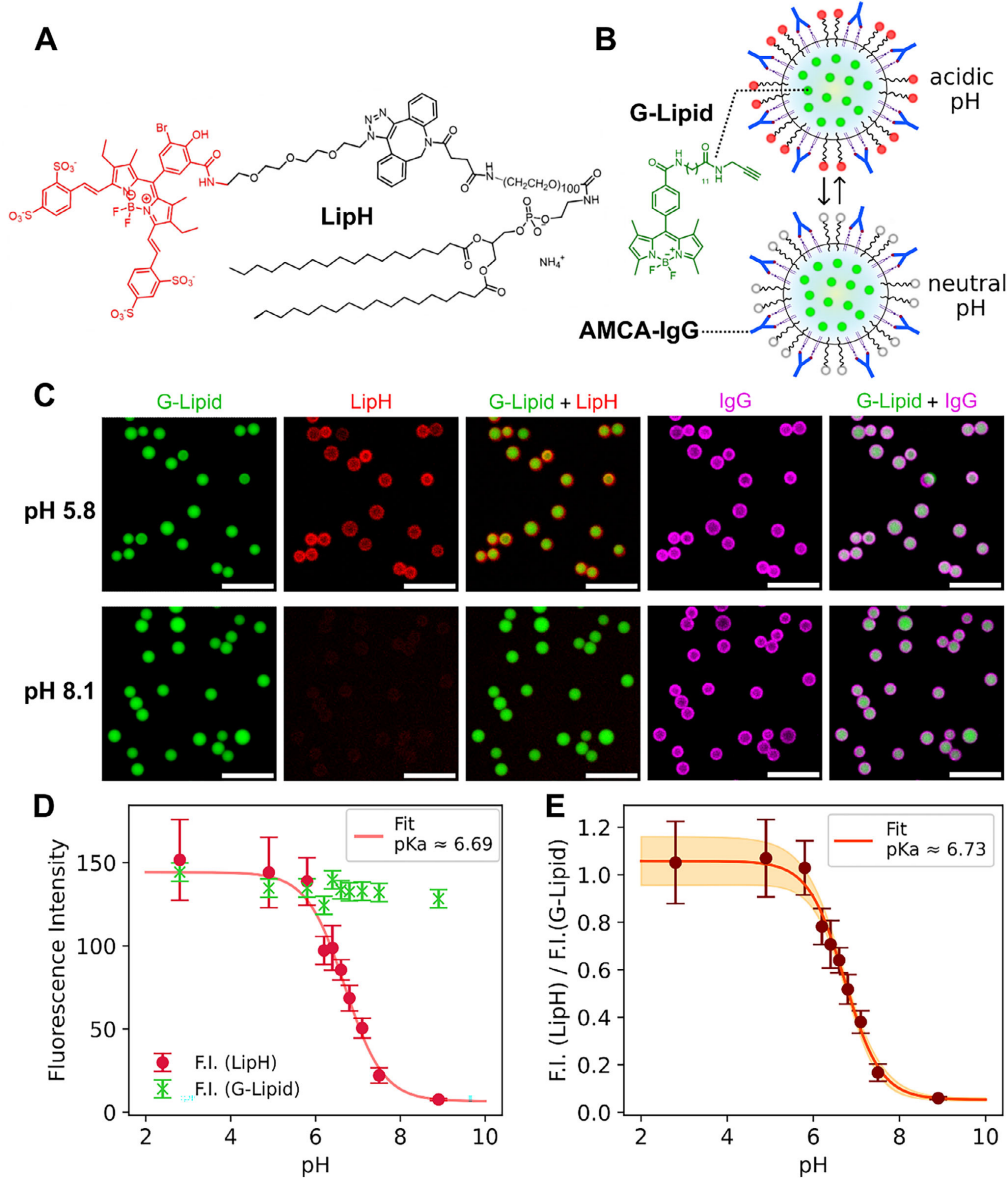
Design and characterization of ratiometric pH‐sensitive droplets. (A) Chemical structure of the fluorescent pH‐sensitive lipid **LipH**. (B) Schematic of the targeted ratiometric droplet design, with LipH and IgG positioned at the surface and a pH‐insensitive reference dye (**G‐lipid**) incorporated in the droplet core. (C) Confocal images of droplets co‐functionalized with G‐Lipid (*λ*_ex_ = 488 nm/*λ*_em_ = 495–555 nm), IgG‐AMCA (*λ*_ex_ = 405 nm/*λ*_em_ = 417–477 nm), and LipH (*λ*_ex_ = 640 nm/*λ*_em_ = 645–725 nm) recorded at two different pH values. Scale bar = 30 µm. (D) Fluorescence intensities in the green (**G‐lipid**) and red (**LipH**) channels of droplets functionalized with **LipH** and **G‐Lipid** depending on pH. For each pH value, the mean intensity + SD of about 500 droplets from two replicates is plotted. The red curve represents a sigmoidal fit of the red channel intensity, yielding a pKa of 6.69. (E) Ratio of the **LipH** fluorescence to the **G‐Lipid** fluorescence as a function of pH. The red curve represents a sigmoidal fit, yielding a pKa of 6.73.

To formulate microparticle sensors, **fr BODIpH 2** was chosen for its sensing properties and far red emission to facilitate multiplexed imaging experiments. Thanks to its azide handle, it was conjugated to a DSPE lipid bearing a dibenzocyclooctyne (DBCO) group via copper‐free azide–alkyne cycloaddition, yielding the amphiphilic compound **LipH** (Figure 3A and Scheme S3). **LipH** includes a long polyethylene glycol PEG_100_ spacer designed to minimize electrostatic interactions between the negatively charged particle surface and the pH‐sensitive fluorophore. In presence of the droplets, the amphiphilic lipid spontaneously localizes at the oil–water interface, forming a stable pH‐responsive fluorescent surface (Figure 3B,C).

To achieve quantitative ratiometric measurements, we introduced a pH‐insensitive reference dye into the droplet core. For this purpose, we used a green emitting dye **G‐lipid—**an intermediate in the synthesis of previously reported fluorescent mannolipids—which, being non‐amphiphilic, preferentially partitions into the oil phase of the droplets (Figure 3B,C) [19]. This internal standard, whose fluorescence remains constant under varying environmental conditions, allows normalization of the intensity changes from the surface‐bound pH sensors.

To specifically engage Fcγ receptors on macrophages and trigger receptor‐mediated phagocytosis, we co‐functionalized 8 µm droplets with LipH, G‐lipid, and IgG tagged with 7‐amino‐4‐methylcoumarin‐3‐acetic acid (AMCA, *λ*_abs_ = 344 nm, *λ*_em_ = 440 nm) following a previously established protocol (Figure 3B) [16].

We calibrated the response of the functionalized droplets in solutions of cell culture medium (RPMI) whose pH were adjusted over a 2–9 range. The fluorescence was measured by epifluorescence microscopy in the far‐red (**LipH**) and green (**G‐lipid**) channels. The functionalized droplets exhibit excellent pH‐sensing behavior closely matching that of the parent probe **fr BODIpH 2**, with a dynamic range showing nearly a 50‐fold increase in fluorescence. The apparent p*K_a_* is however slightly higher on the droplet surface (6.7) than in solution (6.3) leading to a sensing range between pH 5.0 and 8.0 (Figure 3C,D). In contrast, the fluorescence of **G‐lipid** remains essentially constant across the entire pH range, providing a reliable internal reference for constructing calibration curves and enabling quantitative pH measurements (Figure 3E). It is worth noting that the density of LipH on the surface of the particle also slightly affects the pK_a_: shifting from 0.1 surface eq. to 1 surface eq. leads to a pK_a_ decrease of about 0.2 pH unit, likely due to electrostatic repulsion between neighboring lipids that makes deprotonation more difficult (Figure S6). It is thus important to calibrate each batch of droplets with all the co‐functionalizing molecules and lipids.

### 3.3 Application to Quantitative Measurements of pH During Phagocytosis

**FIGURE 4:**
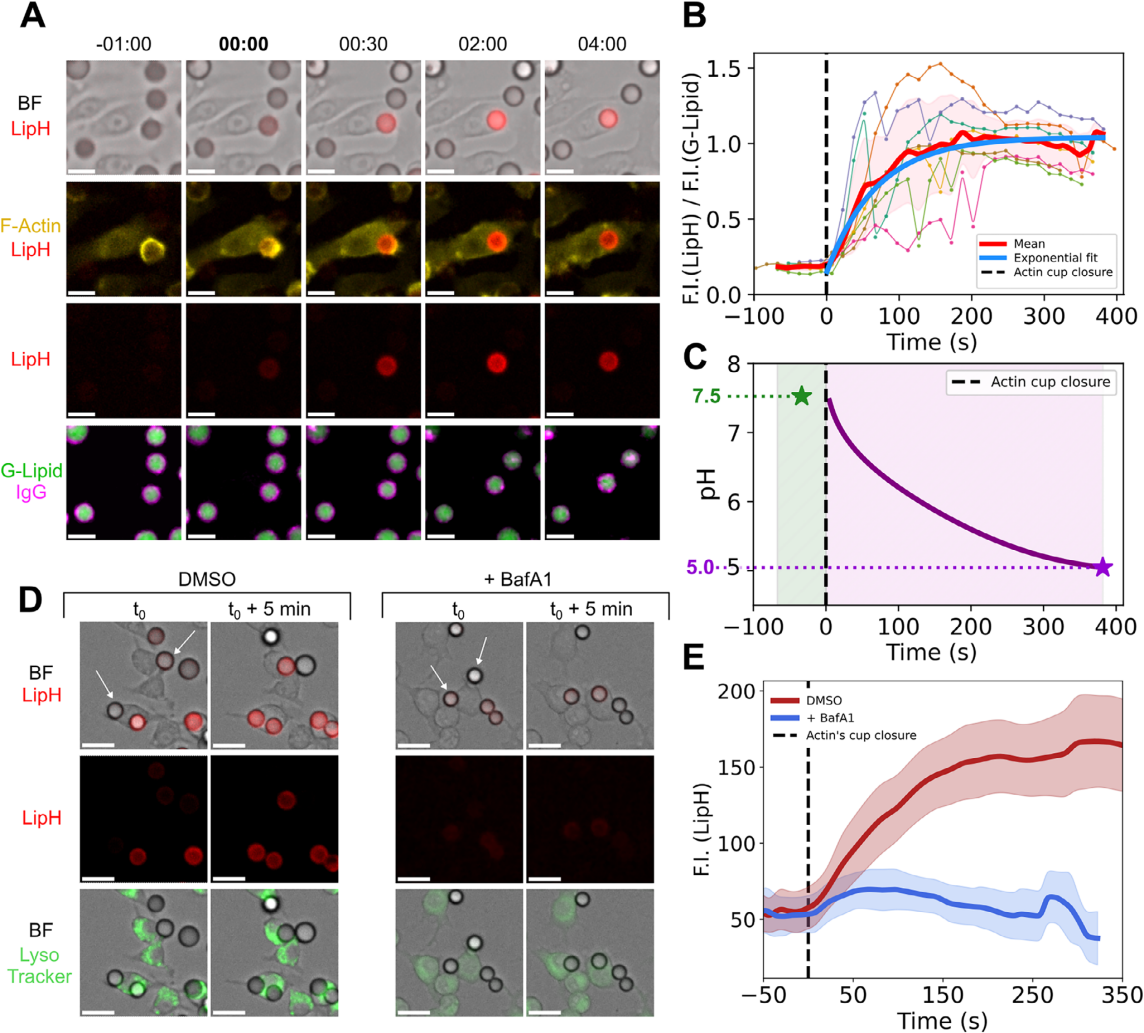
Quantitative phagosomal pH monitoring during the maturation stage. (A) Confocal time‐lapse images of a lipid droplet co‐functionalized with LipH (*λ*_ex_ = 640 nm/*λ*_em_ = 645–725 nm), G‐Lipid (*λ*_ex_ = 488 nm/*λ*_em_ = 495–555 nm), and IgG‐AMCA (*λ*_ex_ = 405 nm/*λ*_em_ = 417–477 nm) during internalization by a RAW264.7 macrophage. The F‐actin channel (*λ*_ex_ = 561 nm/*λ*_em_ = 571–643 nm) marks the closure of the actin cup, indicating the moment of complete engulfment. Scale bar: 10 µm. (B) Time evolution of the LipH/G‐Lipid fluorescence ratio for seven individual droplets during internalization. Traces are aligned to the time of actin cup closure (t = 0 s); the mean + SD of the seven events is shown in red. In blue: exponential fit of the mean ratio after internalization. (C) Time evolution of the pH reported by the probe during internalization. In light green zone: extracellular pH estimated from the mean ratio at t < 0 s. In light pink zone: intracellular pH curve during maturation derived from the exponential fit of the ratio. (D) Confocal images of a lipid droplet functionalized with LipH and IgG in contact with RAW264.7 macrophages. Images were acquired at an initial time t_0_ and at t_0_ + 5 min. Droplets being internalized are indicated with a white arrow. On the left: 1 µL of DMSO was added with the cells for the control. On the right: cells were treated with BafA1 (200 nM) for 4 h to prevent acidification. For both cases: lysosomes were stained with LysoTracker green (100 nM). Scale bar = 15 µm. (E) In blue: time evolution of the mean + SD fluorescence intensity for four individual droplets during internalization when macrophages are treated with BafA1 (200 nM). In red: the mean + SD profile for four individual droplets during internalization by untreated macrophages. Traces are aligned to the time of actin cup closure (t = 0).

We next assessed the ability of these pH biosensors to report phagosomal acidification. When functionalized droplets were presented to RAW 264.7 macrophages, internalized ones appeared markedly brighter than those remaining extracellular (Figure 4A). Quantitative analysis of individual uptake events showed that **LipH** fluorescence increased strongly—by approximately 500%–700%—immediately following closure of the actin cup, revealing that acidification begins as soon as the droplet is fully enclosed (Figure 4B and Movie S1). In some cases, transient fluctuations (“flickering”) precede stabilization (Figure 4B and Figure S7A).

In contrast, G‐lipid fluorescence decreased slightly over time due to photobleaching. This effect was corrected using non‐internalized reference droplets, as detailed in the Supplementary Information and shown in Figure S8. After correction, **G‐lipid** intensity could be considered constant and independent of pH variations (Figure S8B). Consequently, the ratio of **LipH** to **G‐lipid** fluorescence provides a reliable output to quantitatively measure phagosomal pH over time.

The averaged plot of the LipH/G‐lipid fluorescence ratio was fitted with a single‐exponential function, yielding a time constant τ = 69 s that reflects the kinetics of acidification (Figure 4B). Using the calibration curve (Figure 3E), the fitting function was converted to absolute pH values. The phagosomal pH decreased from an initial value of approximately 7.5—consistent with extracellular conditions—to about 5.0 within a little more than six minutes following actin cup closure (Figure 4C)

To confirm that this increase was specifically due to proton accumulation, the main mediator of phagosomal acidification, V‐ATPase, was inhibited with Bafilomycin A1 (BafA1) [3]. Lysosomal fluorescence drastically decreased upon BafA1 treatment, reflecting the loss of acidification in phagosomal and lysosomal compartments (Figure 4D and Figure S9). This decrease confirms that the inhibitor effectively blocked V‐ATPase activity, preventing LysoTracker accumulation [39]. In this case, the droplet fluorescence in the red channel exhibits negligible enhancement after internalization relative to samples without the inhibitor, which supports the specificity of the probe (Figure 4E; Movies S2 and S3).

The kinetics of phagosomal acidification observed in our experiments are consistent with several reports in the literature, although discrepancies remain regarding the precise timing of pH transitions. In our experiments, IgG‐functionalized droplets exhibited a rapid and immediate decrease in intraphagosomal pH, from approximately 7.5 before internalization to ∼5 within less than 10 min after actin cup closure. It should be noted that the dynamic range of our sensor is limited to pH values above 5 and does not allow monitoring the very final stage of phagosome maturation down to pH 4.5. Similar fast kinetics have been reported previously, where phagosomes were shown to acidify immediately following ingestion and to reach a minimal pH of around 5 within 10 – 15 min [7, 8].

However, a recent study describes a three step trajectory: an initial neutral “stand‐by” phase near‐neutral pH, a rapid acidification period over a few minutes, and a plateau at pH 4.5‐5.0 [9]. Our results therefore align with the faster end of this range of kinetics while underscoring the context‐dependence of phagosomal maturation. Our novel pH biosensors provide the ability to precisely tune the particule size and functionalization to engage specific receptors and then follow phagosome maturation with highly dynamic pH probes.

## 4 Conclusion

In this study, we developed a ratiometric pH‐sensing system based on BODIPY‐derived probes incorporated onto biomimetic lipid droplets. Following Fcγ‐receptor specific uptake, they reported rapid phagosomal acidification with a dynamic fluorescence increase of 500%–700% and high temporal resolution. The stabilization of pH within approximately 6 min after actin cup closure indicate that the early stages of phagosome maturation may proceed faster than previously described.

The hydrosoluble BODIpH fluorophores with tunable emissions exhibit excellent pH‐sensing properties well‐suited to monitor acidification during cellular internalization and they could be conjugated in the future to various biomolecules or particles such as dextrans or zymosans to interrogate different endocytic pathways.

Finally, the modularity of the oil‐in‐water droplets system should enable future incorporation of diverse targeting ligands [18, 19], to investigate distinct internalization pathways or additional fluorescent probes to detect other markers of phagosomal maturation such as enzymatic activity.

## Author Contribution

**Sophie Michelis**: Investigation, Synthesis, Data curation, Formal analysis, Visualization, Writing – original draft (figure and data preparation). **Héloïse Uhl**: Investigation, Methodology (phagocytosis and imaging assays), Data curation, Formal analysis, Visualization, Writing – original draft. **Florence Niedergang**: Funding acquisition, Supervision, Writing – review & editing. **Jacques Fattaccioli**: Conceptualization, Supervision, Formal analysis, Visualization, Writing – original draft, Writing—review & editing, Funding acquisition. **Blaise Dumat**: Conceptualization, Supervision, Formal analysis, Visualization, Writing – original draft, Writing – review & editing. **Jean‐Maurice Mallet**: Conceptualization, Supervision, Formal analysis, Writing – review & editing, Funding acquisition.

## Conflicts of Interest

The author declare no conflicts of interest.

## Supporting information



Synthetic procedures, supplementary figures and additional materials and methods are available in the supporting information file. The authors have cited additional references within the Supporting Information [40, 41, 42].

**Movie S1**: Confocal time‐lapse movie of a lipid droplet co‐functionalized with LipH, G‐Lipid, and IgG‐AMCA during its internalization by a RAW264.7 macrophage.

**Movie S2**: Confocal time‐lapse movie of functionalized lipid droplets interacting with RAW264.7 macrophages, whose lysosomes are labeled with LysoTracker green.

**Movie S3**: Confocal time‐lapse movie of functionalized lipid droplets interacting with RAW264.7 macrophages, whose lysosomes are labeled with LysoTracker green in the presence of BafA1.

## Data Availability

The data that supports the findings of this study are available in the supplementary material of this article.
