# Supplementary material for: Ratiometric pH‐Responsive and Biomimetic Microparticles for Quantitative Monitoring of Phagosomal Acidification
Source: Chemistry. 2026 Jun 8;32(35):e71237. doi: 10.1002/chem.71237 (PMC13587647; doi:10.1002/chem.71237)
Supplement: Supplementary file 1 [file CHEM-32-e71237-s002.docx]

**Ratiometric pH-Responsive and Biomimetic Microparticles for Quantitative Monitoring of Phagosomal Acidification**

Sophie Michelis^1,‡^, Héloïse Uhl^1,2,‡^, Florence Niedergang,^3^ Jacques Fattaccioli^1,2^ Blaise Dumat^1^*, Jean-Maurice Mallet^1^*

^1^ Laboratoire CPCV UMR8228, Département de Chimie, École normale supérieure, PSL University, Sorbonne Université, CNRS, 75005 Paris, France

^2^ Institut Pierre-Gilles de Gennes pour la Microfluidique, 75005 Paris, France

^3^ Université Paris Cité, Institut Cochin, INSERM, CNRS, Paris, 75014, France

‡ These authors contributed equally

Corresponding authors : [blaise.dumat@ens.psl.eu](mailto:blaise.dumat@ens.psl.eu); [jean-maurice.mallet@ens.psl.eu](mailto:jean-maurice.mallet@ens.psl.eu)

Table of Contents

[I.	Synthesis of the BodipH and LipH probes	2](#_Toc230268920)

[II.	Additional figures	6](#_Toc230268921)

[III.	Material and methods	12](#_Toc230268922)

[IV.	Synthetic procedures	14](#_Toc230268923)

[V.	NMR and HRMS spectra	18](#_Toc230268924)

[VI.	References	27](#_Toc230268925)

# I. Synthesis of the BodipH and LipH probes

All starting materials for the synthesis were purchased from Sigma-Aldrich, TCI Europe or BLD pharm and used as received. NMR spectra were recorded on a Bruker Advance 300 MHz spectrometer. Mass spectra were obtained using a LTQ-Orbitrap XL (or an Orbitrap Elite for the LipH compound) (Thermo Fisher Scientific, Courtaboeuf, France) and operated in positive or negative ionization mode, with a spray voltage at 3.6 kV. For reactions purposes, all anhydrous solvents were purchased from Sigma-Aldrich.

The synthetic pathway starts from a 3-carboxy-4-hydroxybenzaldehyde precursor (Scheme S1). The acid function was coupled to amino-PEG_3_-azido **2** prior to BODIPY synthesis. Initial synthesis efforts to form the **Yellow** **BODIpH 1** yielded a modest 10% yield with dichloromethane as solvent (procedure 1, Scheme S1). We sought to optimize the procedure for the BODIPY synthesis and obtained good results with the incorporation of ethanol as a co-solvent inspired by a procedure from Radunz *et al*.^[1]^ This minor alteration resulted in a remarkable enhancement of the synthetic yield up to 70 % for **Yellow** **BODIpH 1** (procedure 2, scheme S1).

Scheme S1: Synthesis of yellow BODIpH.

To further decrease the p*K*_a_ of our pH probes, we introduced a bromine atom in the other ortho position of the hydroxyl group. It is recognized that the incorporation of a bromine onto a phenolic structure leads to a reduction of its p*K*_a_ by 1 to 2 units thanks to its attractive inductive effect.^[2,3]^ Bromination was performed on the aldehyde prior to the BODIPY synthesis using N-bromosuccinimide (NBS) to obtain intermediate **4** with 90 % yield. **Yellow BODIpH 2** was then obtained in 45 % yield using the optimized procedure 2 (Scheme S1). It is worth noting that procedure 1 did not allow obtaining **Yellow BODIpH 2** at all, even in low yield.

To tune the emission wavelength and obtain hydrosoluble pH probes, we extended the conjugation on the methyl group in position 3 and 5 using Knoevenagel condensation with 4-formylbenzene-1,3-disulfonate (Scheme S2).

Scheme S2: Synthesis of **Red** **BODipH 1**, **Red** **BODIpH 2**, **fr** **BODIpH 1**, **fr BODIpH 2**. Conditions a, b, c from Table S1.

A procedure reported by Hoogendoorn et al.^[4]^ with ethanol as a solvent and piperidine and acetic acid in large excess using microwave heating at 130°C afforded **Red BODIpH 1** and **fr BODIpH 1** starting from **Yellow BODIpH 1** but the yields were very low and we sought to optimize reaction conditions (Table S1)

Table S1: Overview of the reaction parameters for the coupling with 4-formylbenzene-1,3-disulfonate presented Scheme S2.

|  | Starting material | Solvent | Temperature | Time | Yield |
| --- | --- | --- | --- | --- | --- |
| a | **Yellow BODipH 1** | Ethanol | 130 °C | 20 min | 1% **Red BODIpH 1**  5% **fr BODIpH 1** |
| b | **Yellow BODipH 1** | DMF | r.t. | 24h | 10% **Red BODIpH 1**  23% **fr BODIpH 1** |
| b | **Yellow BODipH 2** | DMF | r.t. and 25 °C | overnight | No product |
| c | **Yellow BODipH 2** | DMSO | 25 °C | 24h | 3% **Red BODIpH 2**  31% **fr BODIpH 2** |

The aldehyde is insoluble in methanol and acetonitrile even at ebullition temperature but seemed to be more soluble in dimethylformamide a solvent in which we managed to reach 33 % of conversion yielding the mono (10 % yield) and di-substituted (23 % yield) products with piperidinium counterions (Table S1). Due to the difficulty to stop the reaction after the mono-substitution, compounds from the **Red BODIpH** series are obtained as intermediary side-products of the bi-substitution with lower yields than for the **fr BODIpH** series. The polar and hydrosoluble products were purified by flash chromatography using a reverse phase C18 column. We performed the same reaction with **Yellow** **BODIpH 2**. Unfortunately, TLC monitoring showed that the reaction did not start even after 24 h using DMF as solvent, probably due to the lack of solubility of **Yellow** **BODIpH 2** in DMF. The latter was replaced by dimethylsulfoxide (DMSO), a solvent in which both reactants are soluble, and the conversion reached 34% at 25°C (Table S1, Scheme S2).

The pH-sensitive fluorescent lipid **LipH** was obtained by copper-free azide alkyne cycloaddition in DMF starting from **fr BodipH 2** and DSPE-DBCO commercial lipid (Avanti research). The product was purified by size exclusion chromatography and the structure analyzed by HRMS.

Scheme S3: Synthesis of **LipH**

# II. Additional figures

Figure S1: Absorbance of **Yellow BODIpH 2** (A), **Red BODIpH 2** (B), **fr BODIpH 2** (C) at varying concentrations in water.

Figure S2. Fluorescence spectra of a 5 µM solution of **Yellow BODIpH 2** in a pH 4.3 aqueous buffer with and without 30 % of acetonitrile.

Figure S3 (A) Absorbance of **Red** **BODIpH 1** in Britton-Robinson buffers, (2 µM). (B) Fluorescence emission of **Red** **BODIpH 1** in Britton-Robinson buffers, (2 µM), λ_ex_= 560 nm (C) Absorbance of **fr** **BODIpH 1** in Britton-Robinson buffer (1 µM) (D) Fluorescence emission of **fr** **BODIpH 1** in Britton-Robinson buffers, λ_ex_= 620 nm (1 µM).

Figure S4: (A) Absorbance of **Red** **BODIpH 2** in Britton-Robinson buffer (5 µM). (D) Fluorescence emission of **Red** **BODipH 2** in Britton-Robinson buffers. λ_ex_ = 560 nm (5 µM). (C) Absorbance of **fr BODIpH 2** in Britton-Robinson buffer (4 µM) (D) Fluorescence emission of **fr** **BODIpH 2** in Britton-Robinson buffers. λ_ex_ = 620 nm (4 µM).

Figure S5. (A) Size distribution of the lipiodol emulsion droplets having a mean diameter of 8.1 ± 0.8 µm. Average of 1337 droplets, determined with fluorescence microscopy. (B) Surface tension measurements made by micropipette aspiration for IgG-functionalized droplets of lipiodol in cell medium (N=18). Measurements yield to γ_lipiodol/med_ = 1.83 ± 0.23 mN/m

| Fitting parameters | R^2^ | pK_a_ |
| --- | --- | --- |
| 0.1 eq | 0.98898 | 6.86±0.10 |
| 0.5 eq | 0.99541 | 6.69±0.04 |
| 1 eq | 0.99866 | 6.63±0.02 |

Figure S6. pH titration of droplets functionalized with 1 eq, 0.5 eq or 0.1 eq **LipH** using a fluorimeter to measure the intensity. Average of 2 normalized experiments on independent functionalized batches. λex= 600 nm. 4.5x106 droplets in 50 μL buffer

 Figure S7. (A) Fluorescence in the **LipH** channel ( λ_ex_ = 640 nm / λ_em_ = 645–725 nm) of 7 different droplets during the process of internalization. (B) Fluorescence in the **G-Lipid** channel (λ_ex_= 488 nm / λ_em_= 495 - 555 nm) of 7 different droplets during the process of internalization.

**Correction of the bleaching fluorescence**

Figure S8: (A) Fluorescence in the **G-Lipid** channel (λ_ex_= 488 nm / λ_em_= 495 - 555 nm) of 7 different droplets during the process of internalization. (B) Fluorescence in the **G-Lipid** channel of 4 different droplets that were not internalized. The microscope settings and analysis routine used are the same as those used for (A). In red, polynomial fit of the mean intensity according time. (C ) Fluorescence in the **G-Lipid** channel of the same 7 droplets as (A) during the process of internalization after the bleaching correction.

To correct the **G-Lipid**’s intensity fluorescence decrease due to bleaching, 4 droplets that remained not internalized were taken into account (Figure S7B). The droplet’s fluorescence intensity in the G-Lipid’s channel (λ_ex_= 488 nm / λ_em_= 495 - 555 nm) was measured for each of them and the mean at each time was measured and fitted by a polynomial function *Bleach(t)* .

The intensity measured for the 7 droplets considered for the study was modified this way :

$${F.I.}_{GLip,corr}\left( t \right)\text{=}\frac{Bleach(0)}{Bleach(t)}\times{F.I.}_{GLip}\left( t \right)$$

Figure S9 : Maximum fluorescence intensity in the green channel (λ_ex_= 488 nm / λ_em_= 495 - 555 nm, corresponding to the LysoTracker emission) measured in individual macrophages incubated with LysoTracker (100 nM) for 20 minutes. Two conditions were analyzed: control cells treated with 1 µL DMSO (in pink, **120 cells were analyzed from two replicates**) and cells treated with BafA1 (200 nM) for 4 hours (in blue, 1**53 cells were analyzed from two replicates**). The same imaging parameters were used for both cases. Each point represents a single cell, and the black diamond indicates the mean ± standard deviation.

**Captions of supplementary movies S1-3:**

**Movie S1**: Confocal time-lapse movie of a lipid droplet co-functionalized with LipH (shown in red, λ_ex = 640 nm, λ_em = 645–725 nm), G-Lipid (shown in green, λ_ex = 488 nm, λ_em = 495–555 nm), and IgG-AMCA (shown in pink; λ_ex = 405 nm, λ_em = 417–477 nm) during its internalization by a RAW264.7 macrophage expressing LifeAct-mCherry (shown in yellow; λ_ex = 561 nm, λ_em = 571–643 nm). After closure of the actin cup, the LipH signal rapidly increases. Scale bar: 10 µm. Time interval: 15 s

**Movie S2**: Confocal time-lapse movie of lipid droplets functionalized with LipH (shown in red, λ_ex = 640 nm, λ_em = 645–725 nm) and IgG interacting with RAW264.7 macrophages, whose lysosomes are labeled with LysoTracker at 100nM (shown in green, λ_ex = 488 nm, λ_em = 495–555 nm) . Cells were incubated with 1 µL DMSO as a control. Scale bar: 15 µm. Time interval: 15 s.

**Movie S3**: Confocal time-lapse movie of lipid droplets functionalized with LipH (shown in red, λ_ex = 640 nm, λ_em = 645–725 nm) and IgG interacting with RAW264.7 macrophages, whose lysosomes are labeled with LysoTracker at 100nM (shown in green, λ_ex = 488 nm, λ_em = 495–555 nm). Cells were treated with BafA1 (200 nM, 4 h) to inhibit acidification. Scale bar: 15 µm. Time interval: 15 s.

# III. Material and methods

**UV-Vis absorption and fluorescence spectroscopies**

Absorption spectra were recorded on a Cary 300 UV-Vis spectrophotometer with a 500 nm/s scan speed and a 1 nm step. Spectra were corrected with a baseline. The extinction coefficients were determined according to the Beer-Lambert law by measuring the absorption of solutions of increasing concentrations.

Emission spectra were recorded on a Jasco FP-8300 spectrofluorometer with a 600 nm/s scan speed and a 1 nm step. The sensitivity was set to medium, and the excitation and emission bandwidths were set to 2.5 nm.

Fluorescence quantum yields were measured by comparison to established reference Rhodamine 6G in ethanol (φ = 0.94).^[5]^ For each dye, the absorbance and fluorescence of five solutions at increasing concentrations were measured. After plotting the curve of the integrated fluorescence intensity against the absorbance at the excitation wavelength (I = f(A)), the quantum yields φ_F_ were calculated using the following equation:

$$\text{ }\phi_{sample}\text{=}{\text{ }\phi}_{ref}\frac{{grad}_{sample}}{{grad}_{ref}}\times\frac{\eta_{sample}^{2}}{\eta_{ref}^{2}}$$

where grad is the slope of the I = f(A) curve and η the refractive index of the solvent.

**Fitting of the fluorimetric titration of the functionalized droplets**

The mean of fluorescence intensity in the LipH channel ( λ_ex_ = 640 nm / λ_em_ = 645–725 nm) and the ratio F.I._LipH_/F.I._G-Lipid_ were plotted according pH and were fitted using a sigmoïd equation:

$$f\left( pH \right)\text{=}\frac{L}{1\text{+}e^{\text{-}k(pH\text{-}pKa)}}\text{+}B$$

yielding the following parameters :

For F.I._LipH_(pH) : L = -137.69 ± 14.98 ; k = 2.21± 0.38 ; pKa = 6.69 ± 0.12 ; B = 144.27 ± 14.48

For F.I._LipH_/F.I._G-Lipid_ (pH) : L = -1.00 ± 0.11 ; k = 2.37 ± 0.42 ; pKa = 6.73 ± 0.11 ; B = 1.06 ± 0.11

The averaged plot of the LipH/G-lipid fluorescence ratio was fitted with a single-exponential function: R(t) = A(1-e^-t/ τ^)+B

yielding the following parameters : A = 0.90 ± 0.01, τ = 69.1 ± 5.9 and B = 0.14 ± 0.01

When timelapses were recorded, a step of 15 seconds was used.

**Image analysis**

Measurements of fluorescence intensity were performed on ImageJ (Fiji Distribution)^[6]^ .

**Fluorescence intensity of LipH and G-Lipid.** For the analysis of fluorescence channels, Z-stacks were combined using an average Z-projection, and background fluorescence was subtracted.

Since droplets were labeled with G-Lipid, their contours were clearly visible, allowing segmentation using a fixed threshold applied to the green channel. The resulting binary mask was used to measure the mean fluorescence intensity in each frame for the red channel (LipH signal) and the green channel (G-Lipid signal). The same binarization threshold was consistently applied to all droplets.

Actin cup closure was determined manually. The last frame in which the droplet appeared partially engulfed was defined as t_last,out_ , and the first frame in which the droplet was fully internalized as t_first,in_ . The time of cup closure was calculated as: t_closure_ =( t_first,in_-t_last,out_)/2.

**Fluorescence intensity of LysoTracker**. For LysoTracker quantification, Z-stacks in the green channel were combined using an average Z-projection, and background fluorescence was removed.

Lysosomal fluorescence was segmented by thresholding the green channel using a constant minimal threshold across all conditions. For each analyzed cell, the maximum fluorescence intensity was measured.

Data analysis and plots were performed with Python (v 3.9.13)

# IV. Synthetic procedures

**Compound 1**

Compound 1 was obtained according to literature with a 86% yield.^[7]^

**^1^H NMR** (300 MHz, Chloroform-*d*) δ 10.04 (s, 1H), 9.91 (s, *J* = 0.6 Hz, 1H), 8.54 (d, *J* = 2.0 Hz, 1H), 8.13 (dd, *J* = 8.7, 2.1 Hz, 1H), 7.19 (d, *J* = 8.7 Hz, 1H), 2.95 (s, 4H).

**Compound 3**

To a solution of **1** (0.300 g, 1.14 mmol, 1 eq) in DMSO (6 mL) under argon was added 2-(2-(2-(2-azidoethoxy)ethoxy)ethoxy)ethan-1-amine (0.298 g, 1.37 mmol, 1.2 eq) and DIEA (0.490 mL, 2.85 mmol, 2.5 eq). The reaction mixture was stirred at r.t. for 4h, then diluted in EtOAc, washed with HCl 1M and extracted (x3). The organic layer was dried over MgSO_4_, filtered and concentrated. The crude product was purified by flash column chromatography (Cyclohexane-EtOAc 60:40 to 40:60). The mixture was concentrated to obtain **3** as orange oil (0.253 g, 80 % yield).

**^1^H NMR** (300 MHz, Chloroform-*d*) δ 13.34 (s, 1H), 9.88 (s, 1H), 8.13 (d, *J* = 2.0 Hz, 1H), 7.90 (dd, *J* = 8.6, 2.0 Hz, 1H), 7.45 (s, 1H), 7.09 (d, *J* = 8.6 Hz, 1H), 3.78 – 3.60 (m, 14H), 3.36 (m,2H).**^13^C NMR** (75 MHz, Chloroform-*d*) δ 190.12, 169.40, 167.09, 135.77, 128.22, 127.82, 119.21, 114.71, 70.79, 70.74, 70.60, 70.34, 70.00, 69.31, 50.69, 39.77.**HRMS** (ESI) calculated for C_16_H_21_N_4_O_6_ 365.1467. Found 365.1464

**Yellow BODIpH 1 procedure 2**

To a solution of **3** (0.290 g, 0.79 mmol, 1 eq) in dry DCM (40 mL) and ethanol (3 mL) under argon was added 3-ethyl,2,4-dimethyl-1H-pyrrole (0.195 g, 1.58 mmol, 2 eq). 5 rapid vacuum-argon cycles were performed. TFA was added (9 µL). The reaction mixture was stirred overnight in the dark until completion. Chloranil (0.194 g, 0.79 mmol, 1 eq) was added and the reaction mixture was stirred for 1 hour. The mixture was concentrated under reduced pressure and the product was diluted in dry DCM (30 mL). DIEA (1.3 mL, 7.9 mmol, 10 eq) was added followed by BF_3_OEt_2_ (1.95 mL, 15.8 mmol, 20 eq). The reaction mixture was stirred 1 hour. The reaction mixture was washed with water (x2), extracted with DCM (x2). The organic layer was dried over MgSO_4_, filtered and concentrated. The crude product was purified by a first short flash column chromatography (90:10 DCM-Cyclohexane to DCM) and then Cyclohexane-EtOAc 90:10 to Cyclohexane-EtOAc 60:40. The mixture was concentrated to obtain **Yellow BODIpH 1** as red solid (0.380 g, 70% yield).

**^1^H NMR** (300 MHz, Chloroform-*d*) δ 12.79 (s, 1H), 7.41 (d, *J* = 2.0 Hz, 1H), 7.28 (dd, *J* = 8.5, 2.0 Hz, 1H), 7.22-7.18 (m, 1H), 7.11 (d, *J* = 8.5 Hz, 1H), 3.65-3.50 (m, 8H), 3.46-3.32 (m, 6H), 3.24 (t, *J* = 5.0 Hz, 2H), 2.52 (s, 6H), 2.31 (q, *J* = 7.6 Hz, 4H), 1.38 (s, 6H), 0.99 (t, *J* = 7.5 Hz, 6H). **^13^C NMR** (75 MHz, Chloroform-*d*) δ 169.57, 162.25, 154.03, 138.99, 138.29, 133.92, 132.99, 131.13, 125.69, 119.41, 114.86, 70.68, 70.59, 70.38, 70.14, 69.72, 69.10, 50.61, 39.66, 26.92, 17.05, 14.65, 12.51, 12.22. **HRMS (ESI**) **m/z** [M+H]+: calculated for C_32_H_42_BF_2_N_6_O_5_ 639.3283 Found 639.3288

**Compound 4**

A solution of **3** (O.240 g, 0.655 mmol, 1 eq) in DMF under argon was stirred in an ice bath. NBS (0.117 g, 0.66 mmol, 1.01 eq) was added slowly. The mixture was heated to 70 degrees for 3 hours. The reaction mixture was concentrated under reduced pressure, diluted in DCM and washed with thiosulfate. It was extracted with DCM, dried over MgSO_4_, filtered and concentrated. The crude product was purified by flash chromatography with 0.1% TFA, Cyclohexane-EtOAc 50:50 to 30:70. The mixture was concentrated to obtain **4** as light brown oil (0.259 g, 90 % yield).

**^1^H NMR** (300 MHz, Methanol-*d*_4_) δ 9.70 (s, 1H), 8.19 (d, *J* = 2.0 Hz, 1H), 8.08 (d, *J* = 1.9 Hz, 1H), 3.58 (m, 14H), 3.27 (t, *J* = 4.9 Hz, 2H). **^13^C NMR** (75 MHz, Methanol-*d*_4_) δ 189.56, 179.35, 169.06, 137.37, 129,17, 127.82, 115.83, 113,36, 77.63, 77.20, 76.77, 70.50, 70.42, 70.40, 69.99, 69.92, 69.36, 50.55, 49.73, 49.44, 49.16, 48.87, 48.59, 48.30, 48.02, 39.72. **HRMS** **(ESI) m/z**: [M+Na]+ calculated for C_16_H_21_BrN_4_O_6_Na 467.0537. Found 467.0534

**Yellow BODIpH 2**

To a solution of **4** (0.247 g, 0.55 mmol, 1 eq) ) in dry DCM (35 mL) and ethanol (2.5 mL) under argon was added 3-ethyl, 2,4-dimethyl-1H-pyrrole (0.195 g, 1.58 mmol, 2 eq). 5 rapid vacuum-argon cycles were performed. TFA was added (6 µL). The reaction mixture was stirred overnight in the dark until completion. Chloranil (0.135 g, 0.55 mmol, 1 eq) was added and the reaction mixture was stirred for 1 hour. The mixture was concentrated under reduced pressure and the product was diluted in dry DCM (30 mL). DIEA (0.950 mL, 5.5 mmol, 10 eq) was added followed by BF_3_OEt_2_ (1.36 mL, 11 mmol, 20 eq). The reaction mixture was stirred 1 hour. The reaction mixture was washed with water (x2), extracted with DCM (x2). The organic layer was dried over MgSO_4_, filtered and concentrated. The crude product was purified by flash column chromatography (Cyclohexane-EtOAc 90:10 to Cyclohexane-EtOAc 70:30. The mixture was concentrated to obtain **Yellow** **BODIpH 2** as red solid (0.166 g, 45% yield).

**^1^H NMR** (300 MHz, Chloroform-*d*) δ 13.77 (s, 1H), 7.59 (d, *J* = 1.9 Hz, 1H), 7.57-7.52 (m, 1H), 7.49 (d, *J* = 2.0 Hz, 1H), 3.73 – 3.53 (m, 8H), 3.53 – 3.44 (m, 2H), 3.45 – 3.35 (m, 4H), 3.28 – 3.18 (m, 2H), 2.51 (s, 6H), 2.31 (q, *J* = 7.5 Hz, 4H), 1.42 (d, *J* = 1.4 Hz, 6H), 0.99 (t, *J* = 7.5 Hz, 6H). **^13^C NMR** (75 MHz, Chloroform-*d*) δ 169.09, 159.01, 154.44, 138.12, 137.24, 136.85, 133.22, 130.95, 126.38, 125.18, 115.75, 113.01, 77.47, 77.04, 76.62, 70.61, 70.49, 70.32, 70.05, 69.64, 69.04, 50.60, 39.94, 26.92, 17.05, 14.66, 12.55, 12.48. **HRMS (ESI) m/z:** [M+Na]+ Calculated for C_32_H_42_BBrF_2_N_6_O_5_Na 741.2353. Found 741.235

**Red and fr BODIpH 1**

To a solution of **Yellow BODIpH 1** (0.035 g, 0.055 mmol, 1 eq) in DMF (1 mL) under argon were 4-formylbenzene-1,3-disulfonate (0.102 g, 0.33 mmol, 6 eq), AcOH (0.030 mL, 0.55 mmol, 10 eq), and piperidine (0.055 mL, 0.55 mmol, 10 eq). The reaction mixture was stirred o/n. The reaction was monitored by reverse phase TLC (Water/Acetonitrile 7:3). The reaction mixture was concentrated under vacuum and co-evaporated with toluene and the residue was purified by reverse flash column chromatography (Water/Acetonitrile 94:6 to 85:15 then to 80:20) to obtain **Red BODIpH 1** (5.8 mg, 10 % yield) as a purple solid and **fr** **BODIpH 1** (18.7 mg, 23 %) as blue solid with piperidinium as counterions.

*Red BODipH 1*

**^1^H NMR** (300 MHz, Methanol-*d*_4_) δ 8.52-8.51 (m, 1H), 8.38 (d, *J* = 16.8 Hz, 1H), 7.92 (d, J=1.2 Hz, 2H), 7.80(s, 1H), 7.76 (d, *J* = 16.8 Hz, 1H), 7.35 (dd, *J* = 8.4, 2.2 Hz, 1H), 7.10 (d, *J* = 8.4 Hz, 1H), 3.70 – 3.47 (m, 14H), 3.30-3.27 (m, 2H), 3.15 – 3.06 (m, 8H), 2.75 (q, J=7.2 Hz, 2H), 2.55 (s, 3H), 2-38 (q, J=7.53 Hz, 2H), 1.81 – 1.71 (m, 8H), 1.63 (m, 4H), 1.47 (d, *J* = 4.0 Hz, 6H), 1.19 (t, *J* = 7.4 Hz, 3H), 1.01 (t, *J* = 7.5 Hz, 3H). **^13^C NMR** (75 MHz, Methanol-*d*_4_) δ 168.97, 156.42, 148.15, 143.87, 143.07, 139.53, 139.45, 138.19, 137.24, 134.17, 133.76, 133.49, 132.58, 132.04, 131.64, 127.94, 127.42, 125.60, 125.14, 122.45, 118.51, 116.32, 70.22, 70.15, 69.99, 69.82, 69.62, 69.00, 50.32, 44.37, 39.19, 22.35, 21.66, 17.70, 16.46, 13.57, 11.60, 11.29, 10.78. **HRMS** (**ESI) m/z**: [M] 2^-^ Calculated for C_39_H_45_BF_2_N_6_O_11_S_2_ 443.1330. Found 443.1328.

*fr BODIpH 1*

**^1^H NMR** (300 MHz, Methanol-*d*_4_) δ 8.54-8.48 (m, 4H), 8.07-7.95 (m, 4H), 7.91 –7.79 (m, 3H), 7.43 (dd, *J* = 8.4 Hz, 2.1 Hz, 1H), 7.17 (d, *J* = 8.4 Hz, 1H), 3.74 – 3.48 (m, 14H), 3.31-3.27 (m, 2H), 3.18 – 3.07 (m, 16H), 2.80 (q, *J* = 7.4 Hz, 4H), 1.79 – 1.74 (m, 16H), 1.73 – 1.59 (m, 8H), 1.53 (s, 6H), 1.30 – 1.19 (m, 6H). **^13^C NMR** (75 MHz, Methanol-*d*_4_) δ 170.34, 162.97, 151.62, 145.54, 144.62, 140.82, 140.55, 138.30, 136.27, 134.95, 134.59, 129.51, 128.95, 127.34, 126.73, 126.47, 123.55, 120.12, 117.82, 71.57, 71.51, 71.34, 71.19, 70.97, 70.39, 51.68, 45.73, 40.55, 23.70, 23.02, 19.11, 14.86, 12.37. **HRMS (ESI) m/z:** [M] 4^-^ Calculated for C_46_H_47_BF_2_N_6_O_17_S_4_ 283.0491. Found 283.049.

**Red and fr BODIpH 2**

To a solution of **Yellow BODIpH 2** (0.046 g, 0.064 mmol, 1 eq) in DMSO (1 mL) under argon were 4-formylbenzene-1,3-disulfonate (0.119 g, 0.38 mmol, 6 eq), AcOH (0.040 mL, 0.64 mmol, 10 eq), and piperidine (0.065 mL, 0.64 mmol, 10 eq). The reaction mixture was heated to 25 °C o/n. The reaction was monitored by reverse phase TLC (Water/Acetonitrile 6:4). The reaction mixture was concentrated under vacuum and co-evaporated with toluene and the residue was purified by reverse flash column chromatography (Water/Acetonitrile 95:5 to 85:15 then to 80:20) to obtain **Red** **BODIpH 2** (1.8 mg, 3 % yield) as a purple solid and **fr** **BODIpH 2** (32 mg, 31%) as blue solid with piperidinium as counterions.

*Red BODIpH 2*

**^1^H NMR** (300 MHz, Methanol-*d*_4_) δ 8.54 – 8.52 (m, 1H), 8.39 (d, *J* = 16.8 Hz, 1H), 7.93 – 7.91 (m, 2H), 7.80 – 7.75 (m, 2H), 7.59 – 7.57 (m, 1H), 3.67 – 3.58 (m, 16H), 3.14 – 3.09 (m, 8H), 2.78 – 2.73 (m, 2H), 2.55 (s, 3H), 2.42-2.39 (m, 2H), 1.79 –1.75 (m, 8H), 1.69 – 1.67 (m, 4H), 1.58 – 1.55 (m, 6H), 1.28 – 1.19 (m, 3H), 1.05 – 1.01 (m, 3H). **^13^C NMR** (75 MHz, Methanol-*d*_4_) δ 170.52, 157.90, 149.49, 145.15, 144.37, 140.75, 139.45, 138.62, 137.27, 135.63, 135.16, 133.97, 133.45, 132.95, 128.81, 126.99, 126.49, 123.86, 71.54, 71.48, 71.34, 71.19, 70.97, 70.44, 51.68, 45.73, 40.60, 30.70, 23.71, 23.03, 19.08, 17.84, 14.93, 12.99, 12.88, 12.36. **HRMS** **(ESI) m/z**: [M] 2^-^ Calculated for C_39_H_44_BBrF_2_N_6_O_11_S_2_ 482.0882. Found 482.0882.

*fr BODIpH 2*

**^1^H NMR** (300 MHz, Methanol-*d*_4_) δ 8.58 – 8.45 (m, 4H), 8.05-7.97 (m, 4H), 7.87-7.82 (m, 3H), 7.63 (s, 1H), 3.74 – 3.54 (m, 16H), 3.11 (t, *J* = 5.5 Hz, 16H), 2.81 (q, *J* = 7.2 Hz, 4H), 1.80 – 1.72 (m, 16H), 1.68 – 1.65 (m, 8H), 1.61 (s, 6H), 1.25 (t, *J* = 7.4 Hz, 6H). **^13^C NMR** (75 MHz, Methanol-*d*_4_) δ 169.22, 158.83, 150.64, 144.35, 143.41, 139.23, 137.06, 136.87, 136.77, 135.16, 133.55, 133.47, 127.56, 126.55, 126.41, 126.00, 125.11, 122.10, 116.26, 112.18, 70.23, 70.14, 69.96, 69.76, 69.61, 68.73, 50.32, 48.49, 48.21, 47.93, 47.64, 47.36, 47.08, 46.79, 44.38, 39.44, 22.33, 21.66, 17.75, 13.49, 11.20. **HRMS (ESI) m/z:** [M] 4^-^ Calculated for C_46_H_47_BBrF_2_N_6_O_17_S_4_ 403.7047. Found 403.7045.

**LipH**

DBCO-PEG (5000)- DSPE (1.5 mg, 0.25 µmol) was added to a solution of **fr BodipH 2** (0.75 mg, 0.5 µmol) in DMF under argon. The reaction mixture was stirred overnight. The reaction was monitored by reverse phase TLC (Water/Acetonitrile 1:9). The reaction mixture was concentrated under vacuum and the residue was purified by steric exclusion chromatography column LH20 stationary phase (DCM/MeOH (1:1)) to obtain LipH as a blue solid (2 mg, quant).

**HRMS** **(ESI-) m/z**: Calculated for [C_306_H_539_BBrF_2_N_9_O_128_PS_4_CH_2_]^5-^ 1338.8821. Found 1338.8788. error = 2.5 ppm.

# V. NMR and HRMS spectra

*Compound 2*

r

*Compound 3*

*Yellow BODIpH 1*

*Yellow BODIpH 2*

*Red BODIpH 1*

*fr BODIpH 1*

*Red BODIpH 2*

*fr BODIpH 2*

High resolution mass spectrometry spectra of *LipH*

# VI. References

[1]	S. Radunz, E. Andresen, C. Würth, A. Koerdt, H. R. Tschiche, U. Resch-Genger, *Anal. Chem.* **2019**, *91*, 7756–7764.

[2]	S. Radunz, H. R. Tschiche, D. Moldenhauer, U. Resch-Genger, *Sensors Actuators, B Chem.* **2017**, *251*, 490–494.

[3]	M. Baruah, W. Qin, N. Basarić, W. M. De Borggraeve, N. Boens, *J. Org. Chem.* **2005**, *70*, 4152–4157.

[4]	S. Hoogendoorn, A. E. M. Blom, L. I. Willems, G. A. Van Der Marel, H. S. Overkleeft, *Org. Lett.* **2011**, *13*, 5656–5659.

[5]	A. M. Brouwer, *Pure Appl. Chem.* **2011**, *83*, 2213–2228.

[6]	J. Schindelin, I. Arganda-Carreras, E. Frise, V. Kaynig, M. Longair, T. Pietzsch, S. Preibisch, C. Rueden, S. Saalfeld, B. Schmid, J.-Y. Tinevez, D. J. White, V. Hartenstein, K. Eliceiri, P. Tomancak, A. Cardona, *Nat. Methods* **2012**, *9*, 676–682.

[7]	G. Despras, A. I. Zamaleeva, L. Dardevet, C. Tisseyre, J. G. Magalhaes, C. Garner, M. De Waard, S. Amigorena, A. Feltz, J.-M. Mallet, M. Collot, *Chem. Sci.* **2015**, *6*, 5928–5937.
